# Comparative Allometric Growth of the Mimetic Ephippid Reef Fishes *Chaetodipterus faber* and *Platax orbicularis*

**DOI:** 10.1371/journal.pone.0143838

**Published:** 2015-12-02

**Authors:** Breno Barros, Yoichi Sakai, Pedro H. C. Pereira, Eric Gasset, Vincent Buchet, Moana Maamaatuaiahutapu, Jonathan S. Ready, Yrlan Oliveira, Tommaso Giarrizzo, Marcelo Vallinoto

**Affiliations:** 1 Universidade Federal do Pará - Campus de Bragança. Instituto de Estudos Costeiros—Laboratório de Evolução. Alameda Leandro Ribeiro, s/n, Aldeia, CEP 68600–000 Bragança, Pará, Brazil; 2 Graduate School of Biosphere Sciences, Laboratory of Aquatic Resources, Hiroshima University, Kagamiyama, 1-4-4, 739–0046, Higashi-Hiroshima, Japan; 3 School of Marine and Tropical Biology, James Cook University—JCU Townsville, QLD, 4811, Australia; 4 Ifremer Centre Océanologique du Pacifique, Unité Ressources Marines en Polynésie française—BP 7004, 98719, Taravao, Polynésie française; 5 Direction des Ressources Maritimes et Minières—BP 20, 98713, Papeete, Polynésie française; 6 Universidade Federal do Pará - Laboratório de Biologia Pesqueira—Manejo dos Recursos Aquáticos. Av. Perimetral 2651 Terra Firme 66040170, Belém, PA—Brazil; 7 CIBIO-InBIO, Centro de Investigação em Biodiversidade e Recursos Genéticos, Campus Agrário de Vairão, Universidade do Porto, 4485–661, Vairão, Portugal; California Polytechnic State University, UNITED STATES

## Abstract

Mimesis is a relatively widespread phenomenon among reef fish, but the ontogenetic processes relevant for mimetic associations in fish are still poorly understood. In the present study, the allometric growth of two allopatric leaf-mimetic species of ephippid fishes, *Chaetodipterus faber* from the Atlantic and *Platax orbicularis* from the Indo-Pacific, was analyzed using ten morphological variables. The development of fins was considered owing to the importance of these structures for mimetic behaviors during early life stages. Despite the anatomical and behavioral similarities in both juvenile and adult stages, *C*. *faber* and *P*. *orbicularis* showed distinct patterns of growth. The overall shape of *C*. *faber* transforms from a rounded-shape in mimetic juveniles to a lengthened profile in adults, while in *P*. *orbicularis*, juveniles present an oblong profile including dorsal and anal fins, with relative fin size diminishing while the overall profile grows rounder in adults. Although the two species are closely-related, the present results suggest that growth patterns in *C*. *faber* and *P*. *orbicularis* are different, and are probably independent events in ephippids that have resulted from similar selective processes.

## Introduction

Cryptic mimesis occurs when a species evolves to closely resemble another or an inanimate object and consequently gains some selective advantage [1]. It is a common strategy adopted by a variety of organisms from insects to mammals, reducing predation rates and increasing survival, mostly during juvenile life phases [1–3]. This phenomenon is relatively widespread and has been studied among reef fish, with approximately 60 cases distributed in 16 families [4,5]. However, despite extensive research analysing the ecological and evolutionary implications of mimicry in fishes [6–8], the importance of relative allometric growth patterns for mimetic fish is still poorly understood. More knowledge on the morphological and anatomical development of mimetic fish is critical to better understand how ontogenetic changes may be involved in altering mimetic efficiency between life stages in these species. In the present study, we use the concept of “cryptic mimesis” as a subdivision of camouflage [1].

Morphological and anatomical changes in vertebrates are known to be important descriptors of life stages, where juveniles and adults present different morphologies in the most cases, and are frequently associated with ecological transitions [9]. In reef fishes, such ontogenetic changes can potentially influence; habitat use by the individuals [10]; diet [11]; feeding preferences [12,13]; and aggressiveness [14]. Considering the ecological importance of these morphological and anatomical changes, they are likely to exert direct influence on the degree and efficiency of mimetic behaviors [15,16]. Unpaired fins (i.e.: dorsal and anal fins) are known to play a substantial role in maneuvering [17,18]. However, the possible influence of such morphological changes on behavioral adaptations has never been studied for plant-mimesis in fish.

The family Ephippidae encompasses eight genera and sixteen fish species [19–21]. Within the family several types of protective mimicry are shared by some genera, mostly during the early stages of development [22]. Cryptic mimesis is a common feature known for juvenile ephippids including *Platax orbicularis*, *P*. *teira* and *P*. *boersii* in the Indo-Pacific, and *Chaetodipterus faber* in the Atlantic [5,15,23–25]. Different mimetic mechanisms are also reported from other *Platax* species. Juveniles of *P*. *pinnatus* mimic a turbellarian flatworm both in coloration and body shape [26], while *P*. *batavianus* juveniles have been identified as mimics of crinoids and sea sponges [22].

The Atlantic Spadefish, *C*. *faber*, is the only ephippid species in the Western Atlantic, widely distributed from South Carolina (US) to Southern Brazil [27]. *C*. *faber* is usually described as a marine, reef-associated species [22,28], although in Brazilian coastal waters it has mostly been observed inhabiting estuarine systems that are closely associated with mangrove environments [29,30]. The orbicular batfish *P*. *orbicularis* also has a broad distribution. It is found throughout the Indo-Pacific systems, from the Red Sea and East Africa, northwards to the Tuamoto Islands, southern Japan, and southwards to northern Australia and New Caledonia, and is also associated to coastal environments such as reefs and mangroves [19]. Both species share highly similar juvenile cryptic mimesis, resembling and behaving like dry leaves near the water surface [16]. Although some studies have analyzed the feeding and social ecology of both species, including aspects of mimetic behavior, there is no study focusing on their allometric growth patterns. It is important to consider allometric data as both species suffer changes in shape, behavior and habitat use at specific growth phases[15,16,19,22,28,31,32]. For both species, only juveniles (standard lengths of up to 6cm in *C*. *faber* and 13cm in *P*. *orbicularis*) are leaf mimetic, and depend on unpaired fin morphology to provide similarity with the plant model [16].

In this context, comparative morphometric analyses of the ephippid species *Chaetodipterus faber* and *Platax orbicularis* were performed to assess growth tendencies during the transition of each species between mimetic and non-mimetic life stages. The allometric relationships describing the morphological changes in these species during their growth were investigated, focusing on processes related to dorsal and anal fins, which are crucial for maintaining the leaf mimetic behavior. The hypothesis that morphometric changes develop through homologous growth patterns in the two mimetic fish species was tested. If development of unpaired fins follows similar patterns in both species, it is expected that general body shape of juveniles and adults of each species would group together in similarity analyses.

## Material and Methods

### Sample acquisition and laboratory procedures

Several different sampling methodologies were employed in order to achieve the best efficiency (ease of capture by different methods varies depending on the location, individual size and life stage) and an adequate sample size for statistical analyses. Individuals were obtained using hook-and-line, hand nets, as well as sample acquisition from local markets, and fish farms.

Sampling of *C*. *faber* in Brazil occurred between 2008–2010 under the national biological sampling (ICMBio-SISBIO) license #18963–2 held by B Barros. At that time, no further national ethical requirements existed, as the SISBIO license covered all practical and ethical requirements for capture and manipulation of biological samples prior to deposition in reference collections. These samples included 25 adults (11 females and 14 males) from the Public Fish Market in Bragança, PA, (1° 3.09’ N 46° 45.44’ W, northern Brazil), 25 non-mimetic subadults and 19 mimetic juveniles obtained directly from local fishermen in Curuçá, PA, (0° 39.04’S, 47° 51.78’W, northern Brazil), and 13 subadults and 12 mimetic juveniles obtained by hand netting whilst snorkeling at Caravelas, BA, (17°42’S, 39°14’W northeastern Brazil) [15,29,30,33]. Sampling of *P*. *orbicularis* in Japan occurred between 2004–2006 and in 2011. As there is no national Japanese licensing framework, samples were collected following the “Guidelines for Proper Conduct of Animal Experiments” set out by the Hiroshima University Animal Research Committee, which are based on international ethical standards[34], and only after obtaining local fishermen community verbal permission for sampling young *P*. *orbicularis*. These samples included twelve mimetic juveniles and nine mimetic subadults from Kuchierabu-Jima Island (30° 28’ N, 130° 10’ E, southern Japan). Euthanasia of samples from Brazil and Japan was performed using a stock solution containing 5ml of 95% eugenol in 1L of ethanol, of which 20ml was diluted in each litre of water containing the fish to be euthanized. No euthanized fish was used in any live experimental work prior to the present study. Sampling of *P*. *orbicularis* in French Polynesia was made in 2013 by photography and measurements of 30 live adult specimens from the Ifremer (14 females and 16 males) (French Research Institute for Exploitation of the Sea, 17° 48’ S, 149° 17’ W, southeastern Pacific) breeding ponds. These were carefully manipulated using diluted benzocaine (150g in 1L of ethanol) as an anesthetic, and released back into the breeding tanks. Fish were monitored visually until completely recovered, and no euthanasia procedures were necessary with these samples. All processes in French Polynesia took place under the French Zootechnical and Veterinary Researchlicense #972–1—VM Buchet. This license includes ethical approval for all manipulation and anesthetic techniques applied. For both species, only adult samples were sexed.

We also used images of both juvenile and adult specimens of *P*. *orbicularis* made available from the following museums and collections, in order to reduce unnecessary sampling efforts while increasing and equalizing sample size in each species and for each mimetic stage: Royal Ontario Museum (N = 1 juvenile Voucher ROM 46208, N = 1 subadult ROM 44287, and N = 2 adults, ROM 44286, ROM 68386); Bernice P. Bishop Museum (N = 1 juvenile, Voucher BPBM 20708, and N = 1 adult BPBM 6968); Kagoshima University Museum (N = 1 adult, Voucher KAUM I 17059); Australian Museum (N = 1 juvenile, Voucher AMS I.45367-00). Sex of individuals obtained from museums and collections was not considered; as such information was not available in most cases.

A total of 52 *C*. *faber* (31 mimetic juveniles, mean standard length SL ± SD = 6.42 ± 0.78 cm; 10 non-mimetic subadults, SL = 10.32 ± 0.41 cm; 11 non-mimetic adults, SL = 25.66 ± 1.03 cm) and 44 *P*. *orbicularis* (15 mimetic juveniles, SL = 3.59 ± 0.16 cm; 29 non-mimetic adults, SL = 33.23 ± 1.88 cm) were eventually used in the present study. High resolution digital pictures of the left lateral view of adult individuals of both species were taken over a black background using a stand table with a reference scale of 5cm. Pictures of juveniles were taken similarly, but over a reference scale of 1cm. Artificial light was used in order to avoid shading of morphological structures. Pictures of live specimens of each stage are provided as supporting information (S1 Fig), where (a) represents a leaf-mimetic *C*. *faber*, (b) a non-mimetic subadult *C*. *faber* (d) a non-mimetic adult *C*. *faber*, (d) a mimetic juvenile *P*. *orbicularis*, (e) a mimetic subadult *P*. *orbicularis*, and (f) a non-mimetic adult *P*. *orbicularis*. All pictures by BBarros, except for (c) and (e), as courtesies of Thierry Zysman and Florent Charpin, respectively.

### Data Analyses

**Fig 1:**
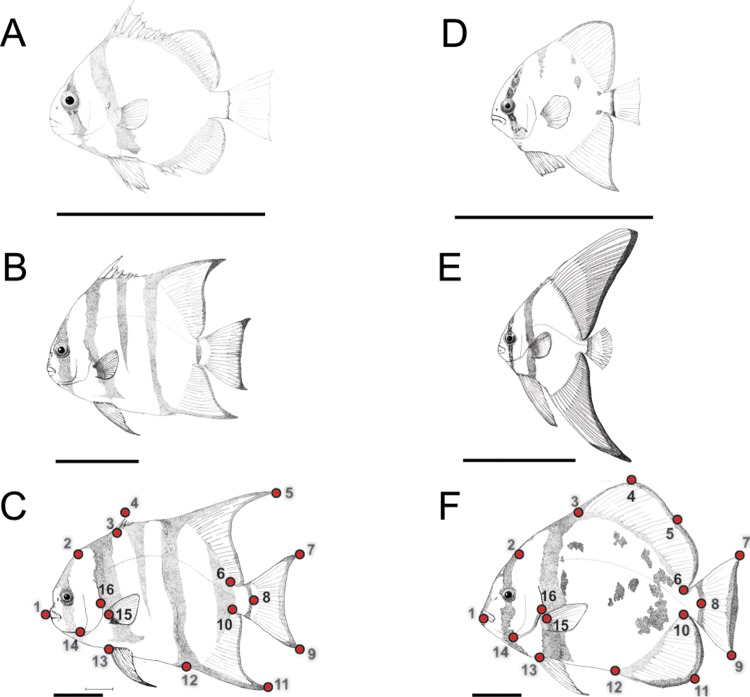
Developmental stages and landmarks of two ephippid fish. Mimetics and non-mimetics of both *C*. *faber* (A-C) and *P*. *orbicularis* (D-F), where (A) shows mimetic juvenile, (B) non-mimetic pre-adult and (C) non-mimetic adult *C*. *faber*; and (D-E) shows mimetic juveniles, and (F) non-mimetic adult *P*. *orbicularis*. Dark bars denote 5cm scale, and dots in adults of both species denote the 16 landmarks used for morphometric measurements in the present study.

For morphometric analysis, a total of 16 homologous landmarks (lm) (Fig 1 and Table 1) and overall body measurements (general body shape, including fins), including body area (BA), were analyzed in each sample using the software ImageJ v.1.47 [35]. The present study specifically requires the inclusion of peripheral reference landmarks (fin extremities) owing to the importance of these features for mimetic behavior. Log centroid sizes (log CS) were obtained from the landmarks, after Generalized Procrustes Analysis (GPA) for each size class within each species, using the software MorphoJ v. 1.02n [36]. In addition to these data, eight other variables were also used for the analysis, including: (1) relative body area (BA/SL); (2) the distance between the edges of dorsal and anal fins (dist lm 5–11); (3) relative distance between the edges of dorsal and anal fins (dist lm 5-11/SL); (4) the angle formed between the edges of the dorsal and anal fins in relation to the fish snout, lm 5-lm1-lm 11 (angle); (5) dorsal fin height, perpendicular distance of lm 5 to midpoint of body outline between lm 3 and lm 6 (df h); (6) relative dorsal fin height (df h/SL); (7) anal fin height, perpendicular distance of lm 11 to midpoint of body outline between lm 10 and lm 12 (af h); and (8) relative anal fin height (af h/SL).

**Table 1:** Description of analyzed landmarks. List of homologous landmarks used in the present study, with the description of each landmark.

Landmark	Description of landmark
1	Tip of the snout
2	Distal limit of supra-occipital
3	Anterior insertion of dorsal fin
4	Distal tip of third spine
5	Distal tip of dorsal fin
6	Posterior insertion of dorsal fin
7	Dorsal tip of caudal fin
8	Posterior limit of urostyle
9	Ventral tip of caudal fin
10	Posterior insertion of anal fin
11	Distal tip of anal fin
12	Anterior insertion of anal fin
13	Anterior insertion of pelvic fin
14	Ventral limit of occipital
15	Dorso-anterior insertion of pectoral fin
16	Posterior limit of occipital process of operculum

The normality of data was assessed both visually (to detect possible outliers) and using the Shapiro-Wilk test (*W* = 0.92 and *W* = 0.90 for juvenile and adult *C*. *faber; W* = 0.89 and *W =* 0.95 for juvenile and adult *P*. *orbicularis*, respectively, *P* > 0.05 for all cases). Bartlett’s test was used to assess homogeneity of variances between each group (Bartlett's K-squared = 2.40, in 1 DF, *P* > 0.05 for juvenile and adult *C*. *faber*, and Bartlett's K-squared = 16.93, 1 DF, *P* > 0.05 for juvenile and adult *P*. *orbicularis*, respectively). Variance among analyzed traits was investigated using a MANOVA test, to assess independence of each measurement per defined group (mimetic vs. non-mimetic for each species). Neither adult *C*. *faber* nor *P*. *orbicularis* show any evidence for variation in morphometric data between males and females (one-way ANOVA with Scheffe’s *post-hoc* test: *F* = 2.26 in 1 and 24 DF, *P* > 0.05 for *C*. *faber*; *F* = 3.09 in 2 and 37 DF, *P* > 0.05 for *P*. *orbicularis*), thus sexual dimorphism was not further considered. Also, no significant variation in morphometric data was observed between subadult (Fig 1B) and adult (Fig 1C) *C*. *faber* (one-way ANOVA with Scheffe’s *post-hoc* test: *F* = 2.14 in 2 and 37 DF, *P* > 0.05), so the latter were placed into a single category for analysis purposes "non-mimetic" (also supported by previous field observations). In comparison *P*. *orbicularis* juveniles (Fig 1D) and subadults (Fig 1E) share mimetic behavior and were grouped together in the single category "mimetic".

Comparisons between mimetic and non-mimetic morphological stages for each species were made with unconstrained ordination of lm data through Principal Components Analysis (PCA) and *post-hoc* ANOVA tests, using the package Geomorph v. 2.0 [37]. The Euclidean distance matrix of lm data were further analyzed using canonical analysis of principal coordinates (CAP) as a constrained ordination and discrimination method, to assess whether there was any significant difference between species (i.e., *C*. *faber/P*. *orbicularis*) and mimetic stages (i.e., mimetic/non-mimetic). The *a priori* hypothesis of four distinct groups (combination of species and mimetic stages) was tested in CAP by obtaining a *P* value using 9999 permutations[38], using PRIMER-E v. 6 with the PERMANOVA add-on [39]. The allometric growth of traits in each species was calculated by means of single regression analysis with ANOVA *post-hoc* tests, confronting each variable against fish standard length (SL: cm). F-statistics are shown followed by degrees of freedom (DF) in all cases. All statistical analyses except for CAP were run in ‘R’ V. 3.2.0 [40]. All necessary data used in the present study are available within supporting material (S1 Dataset).

## Results

Morphometric data varied significantly among the analyzed mimetic classes in both species (MANOVA: Log CS, *F* = 343.94 in 1 and 158 DF; BA/SL, *F* = 7.35 in 1 and 158 DF; angle, *F* = 233.36 in 1 and 158 DF; dist lm 5–11, *F* = 204.54 in 1 and 158 DF; dist 5-11/SL, *F* = 258.75 in 1 and 158 DF; df h, *F* = 243.44 in 1 and 158 DF; dh h/SL, *F* = 103.65 in 1 and 158 DF, af h, *F* = 185.91 in 1 and 158 DF, af h/SL, *F* = 104.65 in 1 and 158 DF. *P* < 0.001 for all cases, Table 2).

**Table 2:** Multiple Analyses of Variance. MANOVA results showing variation in measurements made on mimetic (MIM) and non-mimetic (NMI) individuals of *C*. *faber* and *P*. *orbicularis*. Values are mean values (except F values) with *P* values of < 0.001 separating groups in all cases.

Group	Log CS	BA/SL	angle	dist lm5-11	dist lm5-11/SL	df h	df/SL	af h	af/SL
MIM *C*. *faber*	3.19	2.50	46.08	8.44	0.29	2.84	0.29	2.17	0.22
NMI *C*. *faber*	4.19	0.38	40.62	22.57	0.87	14.20	0.55	11.52	0.45
MIM *P*. *orbicularis*	2.40	0.21	80.25	7.31	2.03	2.58	0.72	2.60	0.71
NMI *P*. *orbicularis*	4.42	2.41	60.91	31.29	0.97	4.23	0.14	5.26	0.16
F	342.94	7.35	233.36	204.54	258.75	243.44	103.65	185.91	104.65
*P*	***	***	***	***	***	***	***	***	***

**Fig 2:**
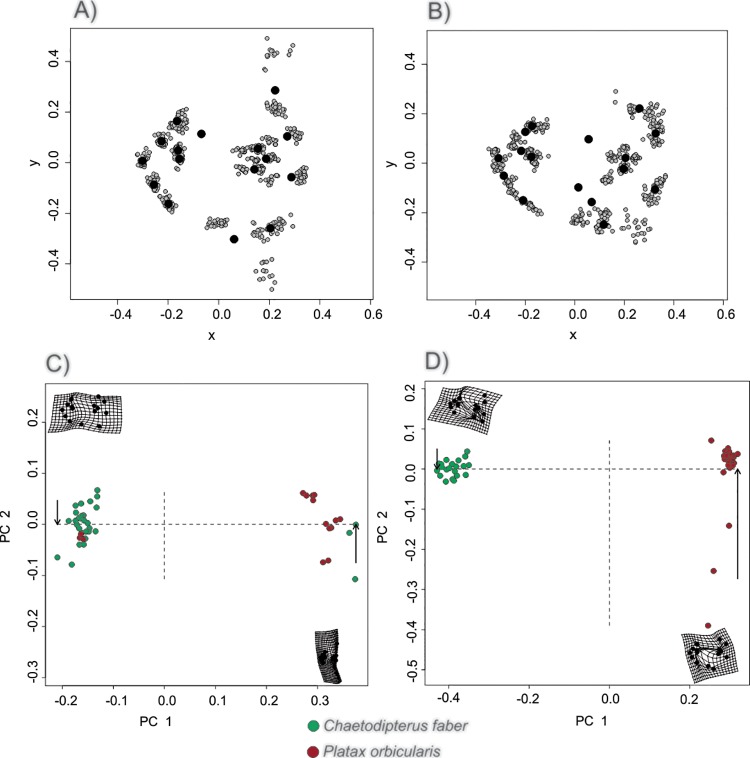
Comparative shape analysis of *C*. *faber* and *P*. *orbicularis*. General shape variations between mimetic and non-mimetic stages for *C*. *faber* and *P*. *orbicularis*, where A) are mimetic individuals of both species and B) are non-mimetic individuals of both species, as analyzed bythe Generalized Procrustes Analysis (GPA). Grey dots represent individual values and black dots represent mean values. Principal Components Analysis (PCA) for mimetic individuals; C) presents a clear distinction in general body shape (small and round in *C*. *faber*; small and oblong in *P*. *orbicularis* (90.73% variance covered by PC1, 2.74% variance covered by PC2), while PCA of non-mimetic individuals of both species; D) shows a tendency to develop a rounded body shape, with greater variation due to inclusion of some elongated individuals (91.03% variance covered by PC1, 4.40% variance covered by PC2). In both cases, green dots represent *C*. *faber* and red dots, *P*. *orbicularis*.

General shape profiles of juveniles do not show great divergence between the two genera, where similar lm distribution patterns were observed in both species during the same mimetic stage, though with most variation observed in lm of unpaired fins (GPA, Fig 2A- Mimetic stage of both species, Fig 2B- Non-mimetic stage of both species). However, PCA indicates that while mimetic stage individuals of both species present a clear distinction between their general shape with little variation within the profile of each species, explained by 90.73% of variance by PC1and 2.74% of variance in PC2 (ANOVA *F* = 379.03in 1 and43 DF; *P* < 0.01; Fig 2C), non-mimetics show greater variation and a tendency to develop a more rounded shape, explained by 91.03% of variance by PC1 and 4.40% of variance in PC2 (ANOVA *F* = 459.05 in 1 and 47 DF; *P* < 0.001; Fig 2D).

**Fig 3:**
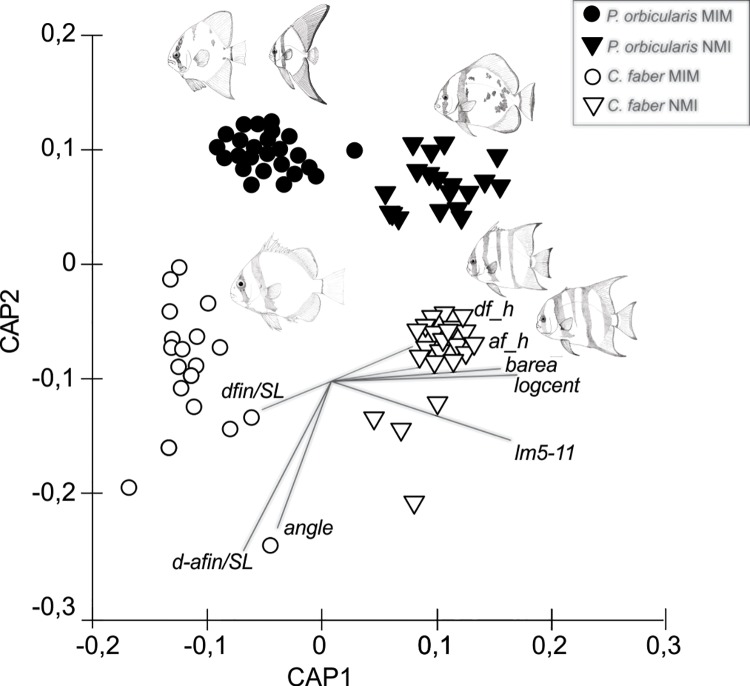
CAP analysis. Constrained canonical analysis of principal coordinates (CAP) ordination plot (Euclidean distance) of mimetic (MIM) and non-mimetic (NMI) individuals of two ephippid fish (*C*. *faber* and *P*. *orbicularis*) based on morphometric measurements data, showing the levels of similarity among groups of mimetic individuals (MIM, circles) and non-mimetic individuals (NMI, triangles), *C*. *faber* (white symbols) and *P*. *orbicularis* (black symbols).

CAP analysis confirmed the separation between species and mimetic stages showing significant differences (**δ** ^2^ = 0.94; *P* = 0.0001). Overall leave-one-out allocation success was 99.01% (i.e., only 0.99% misclassification error) for the combined factors of species and mimetic stage. More specifically, 100% of mimetic and non-mimetic individuals of *P*. *orbicularis* and non-mimetic *C*. *faber* were correctly allocated, while 96.7% of mimetic individuals of *C*. *faber* were correctly classified. The first canonical axis (CAP1) separated the mimetic and non-mimetic specimens, and the second axis (CAP2) the species (Fig 3).

**Fig 4:**
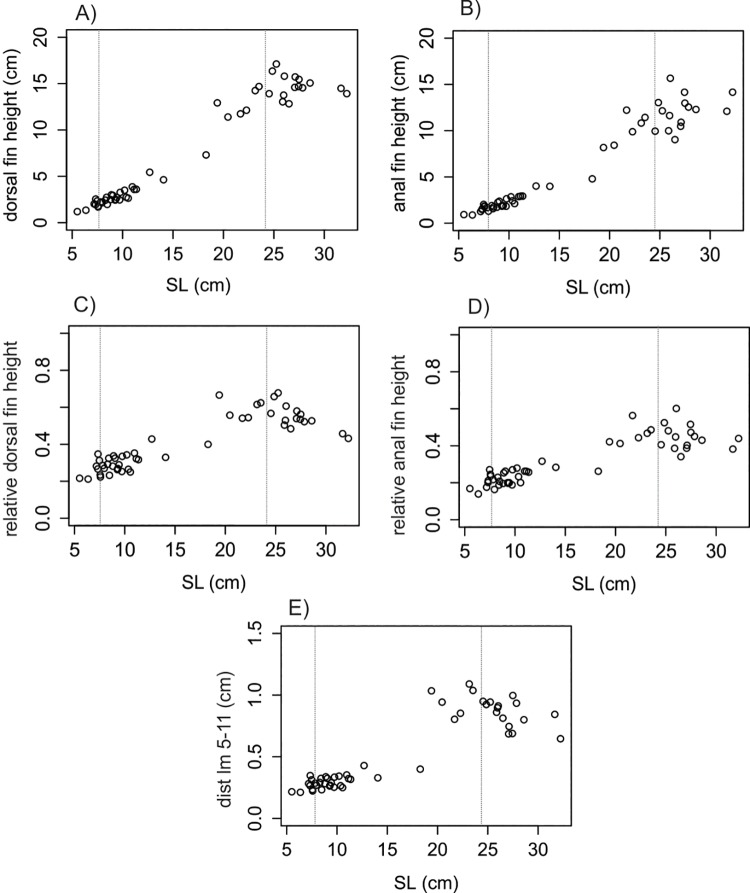
Linear analyses of fins in *C*. *faber*. Allometric relationships of dorsal and anal fins of *C*. *faber* represented by single linear regression: A) Dorsal fin height regressed against SL; B) Anal fin height regressed against SL; C) Relative height of dorsal fin regressed against SL; D) Relative height of anal fin regressed against SL; E) Distance between the edges of dorsal and anal fins regressed against SL. Vertical dotted lines indicate transition between mimetic juvenile—subadults (ca. 8 cm) and non-mimetic subadults to non-mimetic adults (ca. 25 cm).

**Fig 5:**
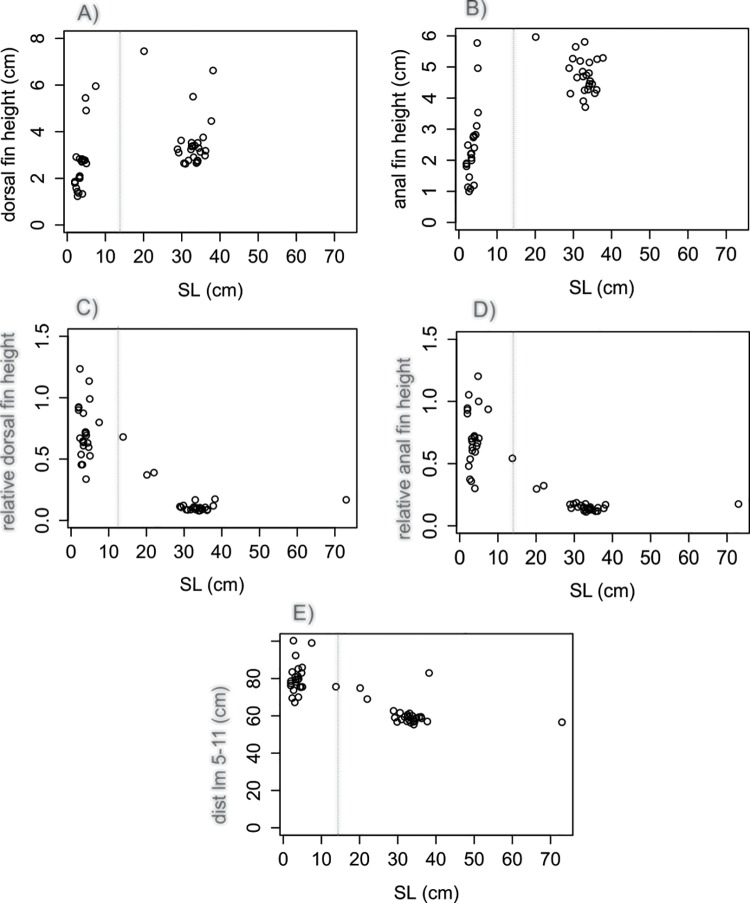
Linear analyses of fins in *P*. *orbicularis*. Allometric relationships of dorsal and anal fins of *P*. *orbicularis* represented by single linear regression: A) Dorsal fin height regressed against SL; B) Anal fin height regressed against SL; C) Relative height of dorsal fin regressed against SL; D) Relative height of anal fin regressed against SL; E) Distance between the edges of dorsal and anal fins regressed against SL. Vertical dotted lines indicate transition between mimetic—non-mimetic (ca. 14 cm).

**Fig 6:**
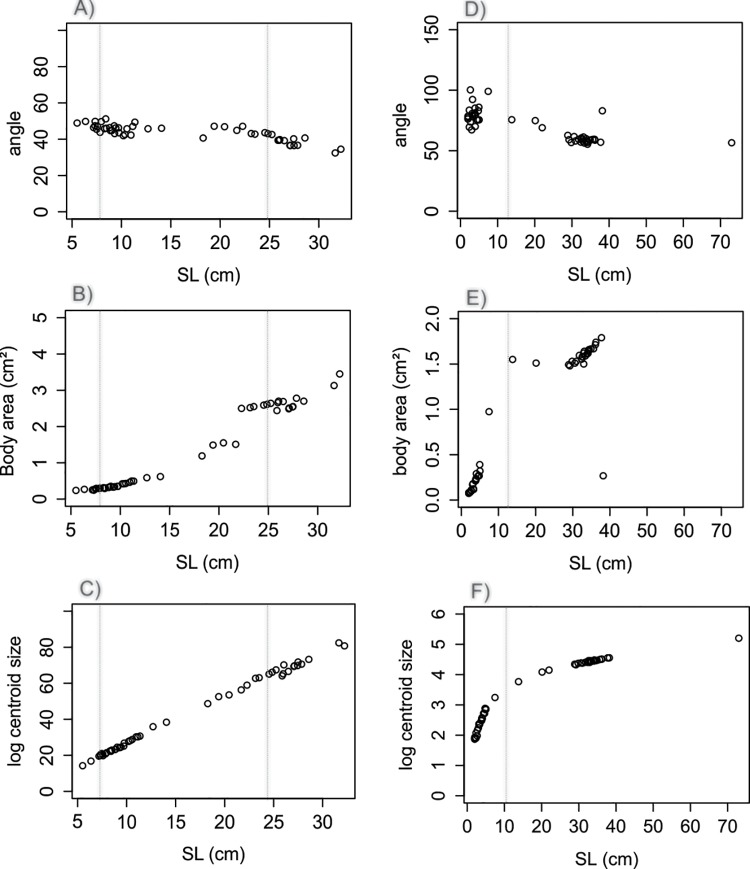
Comparative linear analyses in *C*. *faber* and *P*. *orbicularis*. Comparative allometric relationships between *C*. *faber* and *P*. *orbicularis*: angle formed between the edges of dorsal and anal fins regressed against SL (A—*C*. *faber*, D—*P*. *orbicularis*); fish body area regressed against SL (B—*C*. *faber*, E—*P*. *orbicularis*); log centroid size regressed against SL (C—*C*. *faber*, F—*P*. *orbicularis*). Vertical dotted lines indicate transitions among growth stages (ca. SL 8cm between leaf-mimetic and subadults, and SL ca. 25cm between subadults and adults in *C*. *faber*; ca. SL 12cm between leaf-mimetic and non-mimetic in *P*. *orbicularis*).

Single linear regression analysis confronting all variables against SL revealed different allometric relationships between traits in each species (Figs 4–6). The relationships between dorsal and anal fins heights and SL were observed to follow a linear pattern for *C*. *faber* (df h: *F* = 828.7 in 1 and 49 DF, R^2^ = 0.94, *P* < 0.001,Fig 4A; af h: *F* = 747 in 1 and 49 DF, R^2^ = 0.93, *P* < 0.001, Fig 4B), while the same patterns were not observed for *P*. *orbicularis* (df h: *F* = 13.51 in 1 and 51 DF, R^2^ = 0.19, *P* < 0.001, Fig 5A; af h: *F* = 60.42 in 1 and 51 DF, R^2^ = 0.53, *P* < 0.001 Fig 5B). A linear pattern was observed for the relative height of dorsal and anal fins, but with different sign in each species. *C*. *faber* presented a positive allometry (df h/SL: *F* = 142.12 in 1 and 49 DF, R^2^ = 0.73, *P* > 0.001, Fig 4C; af h/SL: *F* = 164.97 in 1 and 49 DF, R^2^ = 0.77, *P* < 0.001, Fig 4D), while *P*. *orbicularis* presented negative allometry (df h/SL: *F* = 119.33 in 1 and 51 DF, R^2^ = 0.69, *P* < 0.001, Fig 5C; af h/SL: *F* = 102.3 in 1 and 51 DF, R^2^ = 0.66, *P* < 0.001, Fig 5D). A similar pattern of opposite allometry is observed for the relative distance between the edges of dorsal and anal fins and SL in each species (*C*. *faber*, dist lm 5-11/SL: *F* = 188.42 in 1 and 49 DF, R^2^ = 0.78, *P* < 0.001, Fig 4E; *P*. *orbicularis*, dist lm 5-11/SL: *F* = 82.42 in 1 and 51 DF, R^2^ = 0.61, *P* < 0.001, Fig 5E).

The angle formed between the edges of unpaired fins presented a similar allometric growth patterns in both species, where a slightly negative slope was observed (*C*. *faber*, angle: *F* = 82.24 in 1 and 49 DF, R^2^ = 0.62, *P* < 0.001, Fig 6A; *P*. *orbicularis*, angle: *F* = 82.43 in 1 and 51 DF, R^2^ = 0.61, *P* < 0.001, Fig 6D).

Body area presented different allometric growth patterns in *C*. *faber* and *P*. *orbicularis*, with a positive slope in *C*. *faber* (BA: *F* = 1541 in 1 and 49 DF, R^2^ = 0.96, *P* < 0.001, Fig 6B) and no directional trend in *P*. *orbicularis* allometric growth (BA: *F* = 30.87 in 1 and 51 DF, R^2^ = 0.36, *P* < 0.001, Fig 6E).

Log centroid size showed similar directionality for allometric growth in both species (*C*. *faber*, log CS: *F* = 1523 in 1 and 49 DF, R^2^ = 0.99, *P* < 0.001 Fig 6C; *P*. *orbicularis*, log CS: *F* = 433.23 in 1 and 51 DF, R^2^ = 0.89, *P* < 0.001, Fig 6F), but with accelerated early growth and subsequent slower growth in *P*. *orbicularis*.

## Discussion

Cavalluzzi [41], based in osteological data, has proposed that both *Chaetodipterus* and *Platax* genera are monophyletic, with *Chaetodipterus* the most basal ephippid, followed by the genera *Ephippus*, *Tripterodon*, *Zabidius* and *Platax* suggesting that the evolution of leaf mimesis may have evolved in distinct phylogenetic lineages. On the other hand, Tang et al. [42] have shown a very close relationship of the clade *Chaetopdipterus* + *Platax* among other Acanthuroidei fish, based on both molecular and morphological data. Therefore, as cryptic mimesis or mimicry of other organisms is a common trend within ephippids, there may be a connection between the mimetic capabilities of these species. Whether similarities in adaptation depend more on phylogenetic proximity or on similarity of the environments in which individual species are found will require a more complete analysis of additional ephippid species. Although both *C*. *faber* and *P*. *orbicularis* resemble similar floating leaf models during their juvenile life phase [16,23,24], and share similar latitudinal coastal distributions in the Atlantic and Indo-Pacific Oceans [19,27] the results from our study showed that despite these ecological and behavioral similarities, and regardless of how closely related the species are, the allometric growth of both species is an independent process. Also, despite the similarities, the use of morphometric data within different mimetic and non-mimetic classes allows both species to be reliably classified using traits that relate to dorsal and anal fin morphology.

### Allometric growth of dorsal and anal fins

Ditty et al. [43] have observed that unpaired fins start to develop very early during the flexion larval stage of *C*. *faber*, where the development of dorsal and anal fin bases coincides with notochord flexion. Observations in *P*. *orbicularis* show that these fins greatly elongate (along with pelvic fins) during the larval to juvenile transition, giving juvenile fish a "bat-like" appearance [44]. However, this is not observed in early larval stages of the Atlantic species *C*. *faber* which, in contrast, present disproportional elongation of the third spine of the dorsal fin when compared to the general fin shape [43,45]. This is evident for individuals up to a given size (as observed in non-mimetic subadults, 15-25cm), probably related to the end of the growth stage.

Dorsal and anal fins appear to be closely related to leaf-mimesis behavior for both *C*. *faber* and *P*. *orbicularis* [15,16], and also for other mimetic fish species [23]. Another leaf-resembling species, the freshwater Amazonian leaf fish *Monocirrhus polyacanthus* also shows a fast development of unpaired fins [46], which confer the mimetic capacity in this species [23,47,48]. Dorsal and anal fins are furthermore known to be important structures for fish body balance during maneuvering [17,18], and are critical for floating or drifting movements when imitating plant material [15]. Consequently, a fast-differentiated growth of body structures such as dorsal and anal fins in these fish is likely to be relevant as an early step to adapt to the pelagic environment.

### Allometric growth vs. changes in habitat use

Changes in habitat use are important for marine fishes and are usually associated with a combination of morphological and behavioral alterations including changes in diet, social behavior and spatial use of the water column [12,14,49–51]. However, the majority of studies that focus on changes in habitat use have only considered the morphological and morphometric variation of adults of different species rather than considering different life stages. Changes in body shape usually lead to changes in individual habitat use [32,52–54]. Indeed, Loy et al. [55, 56] observed that morphometric changes in early stages of sea bream (Sparidae) are important for different species settlement. The transition from pelagic to benthic environments is defined by morphological variation related to swimming capacity and feeding behavior. The present study suggests that allometric growth of certain traits in both species may present relevant clues to understand changes in habitat use that co-occur with the transition from mimetic to non-mimetic life stages. Specifically, as both species change from shallow, coastal environments to open, deep waters, significant changes in fin morphometry correlated to fish size were observed in the present study. This trend was particularly notable for the orbicular batfish *P*. *orbicularis*, where distinct dorsal and anal fin growth patterns were observed between mimetic and non-mimetic individuals. In contrast, for *C*. *faber* the same characters gradually elongate with respect to fish growth, following a positive allometric relationship. Considering unpaired fin morphology, only the distance between the edges of dorsal and anal fins, and the angle formed by them were observed to follow a similar allometric growth pattern in both species. An increase in the distance between the edges of unpaired fins is expected with fish growth, associated with an increase in individual body area [52]. Accordingly, during the present study we observed that *P*. *orbicularis* presented a growth pattern with a decrease in body height, from an oblong profile in mimetic individuals to a rounded shape in non-mimetic individuals. In contrast, *C*. *faber* presented an opposite trend with small and round mimetic individuals lengthening in the transition to the non-mimetic stage. Although generally similar, any differences in the ecosystems that these species normally occupy during the mimetic stage (plant models, light environments and possible predators) may help to explain the observed different growth patterns. *P*. *orbicularis* mimetic stages present a deeper body, with tall fins that might contribute for behaving like a drifting leaf and therefore reduce predation risk from visual predators [15]. The rounded profile of *C*. *faber* mimetic stages resembles a drifting mangrove leaf, the longer profile in the non-mimetic stage may make it easier to dash during encounters with possible predators in turbid coastal waters (unpublished data).

Barros et al. [32] observed a negative relationship between fish size and feeding behavior related to zooplanktivory in *P*. *orbicularis*. This indicates that behavioral feeding changes occur in very early development stages, even across a small size range, where juvenile fish tend to behave more similarly to settled adults during feeding behavior. In addition, Leis et al. [31] compared the swimming performance, compass orientation and depth preferences during the release of artificially reared young *P*. *orbicularis* (1.7–7.5 cm), observing an ontogenetic descent, with small individuals concentrating activities near to the surface, where such mimetic juveniles are usually distributed in nature, intermediate sizes in mid-water, and bigger individuals tending to descend to the bottom. These authors suggested several intermediate settlement steps in the *P*. *orbicularis* ontogeny, but with some responses that are also independent of fish size, such as swimming speed and orientation. Although reared juvenile *P*. *orbicularis* present slight differences in the shape of unpaired fins when compared to wild juveniles [57], the observations of Leis et al. [31] may be important for inferring the importance of changes in early behavior of larvae and juveniles under ecological and behavioral selective processes, yet most observations so far have shown mimetic *P*. *orbicularis* dwelling near-surface environments [15,16,32].

Atlantic spadefish *C*. *faber* also present habitat changes associated with behavioral modifications during ontogeny, although much less is known concerning leaf mimesis in this species. This species also presents drift swimming behavior in the mimetic stage; often laying over on one side of the body in shallow coastal waters, and are frequently solitary [16,23,24]. Non-mimetics behave differently, living in deeper environments and usually forming aggregations [27,29,30,58].

## Conclusions

In the present study, data regarding growth of *C*. *faber* and *P*. *orbicularis* was analyzed, especially focusing on the allometric growth of unpaired fins, which are considered to be important for changes in habitat use and behavior. The results are relevant to understand mimetic behavioral changes related to different body shapes during different life stages. The ecological and evolutionary importance of mimicry in reef fish communities has already been demonstrated [4,5]. However, there are still many gaps in our knowledge about leaf mimesis, with phenomena that need to be experimentally tested through field and laboratory research. The present data suggests that the processes leading to such morphological changes may have evolved as independent events in each species, with similar ecological and behavioral implications.

## Supporting Information

S1 Dataset Dataset containing all relevant data for the analyses in the present study. (CSV)

S1 Fig Live specimens of (a) mimetic, (b) subadult and (c) adult *Chaetodipterus faber* and (e) mimetic, (f) subadult and (g) adult *Platax orbicularis*. (JPG)
