# Supplementary figures and images for: Comparative Allometric Growth of the Mimetic Ephippid Reef Fishes Chaetodipterus faber and Platax orbicularis
Source: PLoS One. 2015 Dec 2;10(12):e0143838. doi: 10.1371/journal.pone.0143838 (PMC4668021; doi:10.1371/journal.pone.0143838)

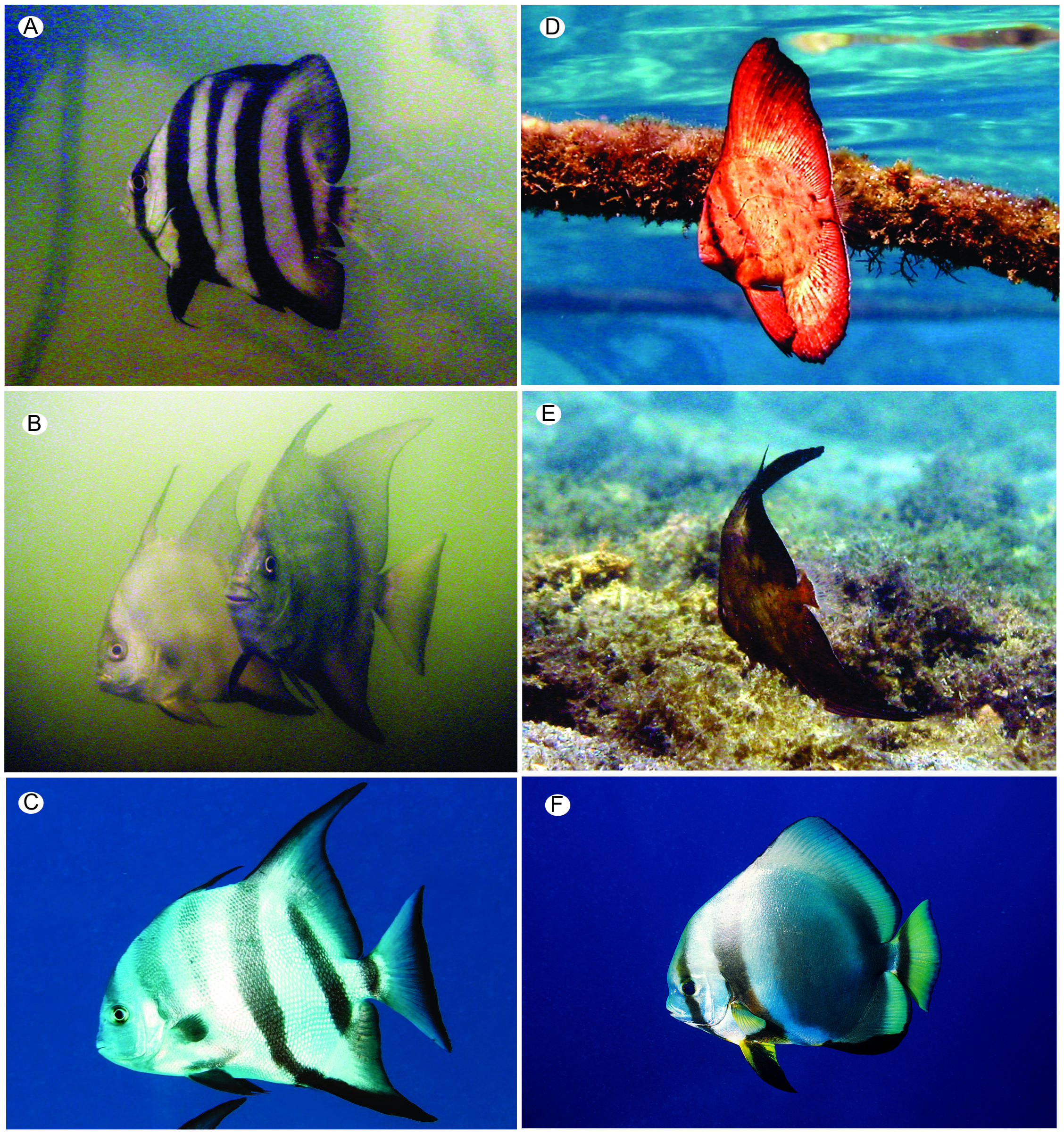

Supplement: S1 Fig — (JPG) [file pone.0143838.s002.jpg]
